# The cardio-renal-metabolic connection: a review of the evidence

**DOI:** 10.1186/s12933-023-01937-x

**Published:** 2023-07-31

**Authors:** Marella Marassi, Gian Paolo Fadini

**Affiliations:** 1 Department of Medicine, Division of Metabolic Diseases, University of Padova, Via Giustiniani 2, 35128 Padua, Italy; 2 Veneto Institute of Molecular Medicine, 35129 Padua, Italy

**Keywords:** Metabolic syndrome, Comorbidities, Cardiorenal, Cardiometabolic, Pathophysiology, Epidemiology

## Abstract

Type 2 diabetes (T2D), cardiovascular disease (CVD) and chronic kidney disease (CKD), are recognized among the most disruptive public health issues of the current century. A large body of evidence from epidemiological and clinical research supports the existence of a strong interconnection between these conditions, such that the unifying term cardio-metabolic-renal (CMR) disease has been defined. This coexistence has remarkable epidemiological, pathophysiologic, and prognostic implications. The mechanisms of hyperglycemia-induced damage to the cardio-renal system are well validated, as are those that tie cardiac and renal disease together. Yet, it remains controversial how and to what extent CVD and CKD can promote metabolic dysregulation. The aim of this review is to recapitulate the epidemiology of the CMR connections; to discuss the well-established, as well as the putative and emerging mechanisms implicated in the interplay among these three entities; and to provide a pathophysiological background for an integrated therapeutic intervention aiming at interrupting this vicious crosstalks.

## Introduction

The International Diabetes Federation estimates that there are currently more than 500 million people living with diabetes worldwide, the vast majority of whom suffering from type 2 diabetes (T2D) [1]. Moreover, globally, there are about 64 million persons diagnosed with heart failure (HF) [2] and almost 700 million individuals affected by chronic kidney disease (CKD) [3], these three entities being the major pandemics of the twenty-first century. Taken individually, each of these three conditions are associated with relevant morbidity and mortality, but it is broadly recognized that they often coexist: patients with HF have a four-fold higher prevalence of T2D (20%) than patients without HF (4–6%) [4], and T2D is associated with a two-to four-fold higher risk of developing cardiovascular disease (CVD) [5]. Furthermore, recent statistics report a CKD prevalence close to 40% among individuals with T2D [6] and 50% among individuals with HF [7]. Conversely, CVD is diagnosed more frequently among patients with CKD than in the general population, being its prevalence inversely related with kidney function [8].

**Fig. 1:**
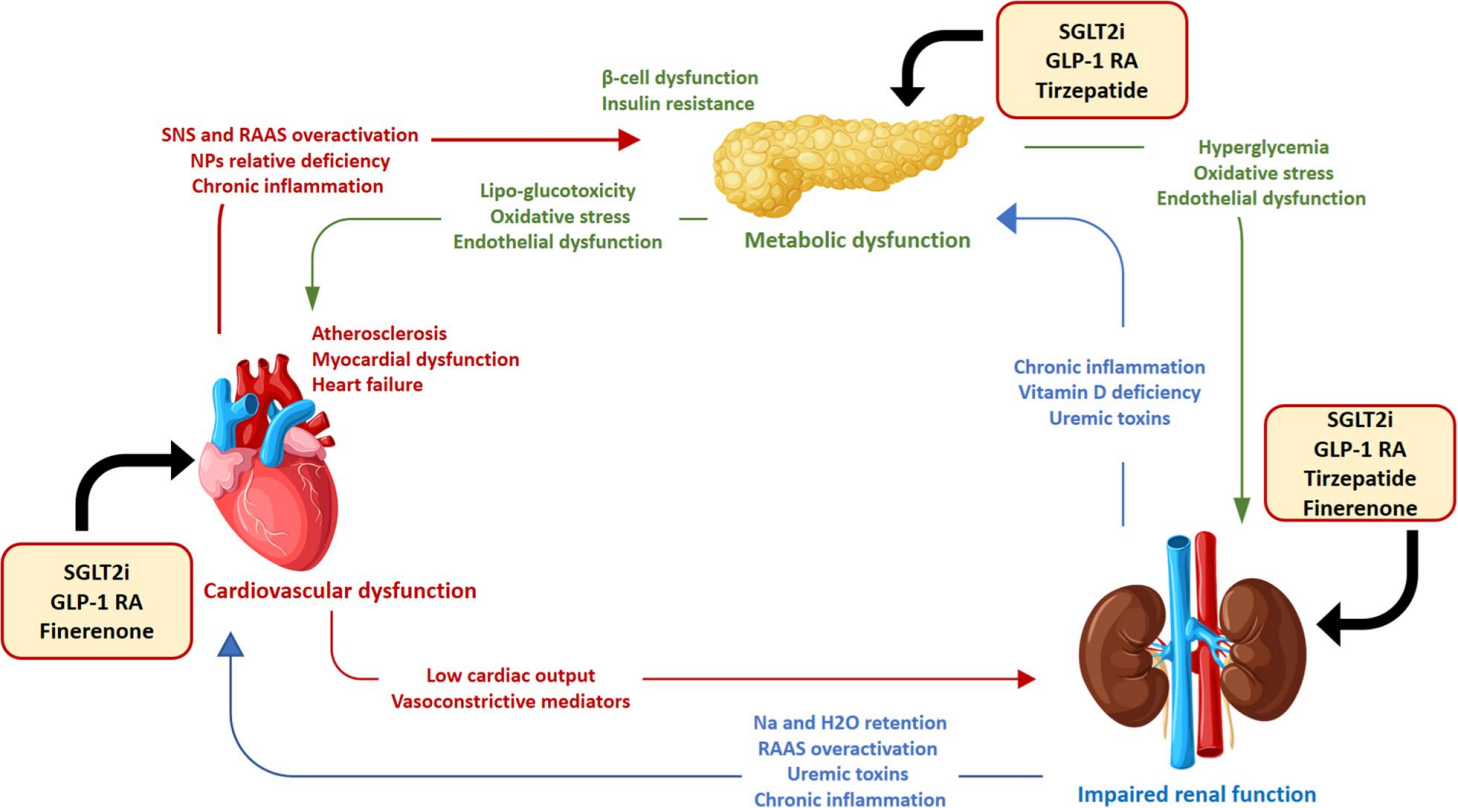
Cardio-metabolic-renal interconnections and therapeutic options. SNS, sympathetic nervous system; RAAS, renin–angiotensin–aldosterone system; NPs, natriuretic peptides; SGLT2i: sodium-glucose cotransporter 2 inhibitors; GLP1- RA: glucagon-like peptide 1 receptor agonists

Since growing evidence supports the existence of a strong interplay among T2D, CVD and CKD, the unifying term cardio-metabolic-renal (CMR) disease has been introduced to describe the systemic interdependence of these conditions [9] (Fig. 1).

While the mechanisms whereby T2D promotes the onset of CVD and CKD have been widely described in the literature, how to what extent HF and CKD promote the development of T2D or worsen T2D control is still much less appreciated. The aim of this review is to summarize the current knowledge of the multidirectional pathophysiological interactions that occur among these three entities.

## Epidemiology of the cardio-renal-metabolic disease

Epidemiological studies have consistently demonstrated that cardiovascular, renal, and metabolic diseases often overlap and coexist in the same patients. According to a meta-analysis of 102 prospective studies, individuals with T2D have a two-fold higher risk for coronary heart disease, stroke and death attributed to other vascular causes [10]. In the study by Kodama et al., T2D was a significant risk factor for both new-onset (risk ratio [RR], 2.14 [95% CI 1.96–2.34]) and recurrent HF (RR, 1.39 [95% CI 1.33–1.45]), and the risk of HF associated with T2D was increased especially in young populations [11]. Conversely, T2D prevalence is greater in HF cohorts than in the general population, with reports of 24% among overall patients with HF and of 40% among those hospitalized with worsening HF [12]. Individuals with HF have an increased risk for subsequent onset of T2D after adjustment for multiple cardiovascular confounders [13], and an analysis from the Candesartan in Heart Failure Assessment of Reduction in Mortality and Morbidity (CHARM) program reported an incidence of T2D of 28 per 1000 person-years among initially nondiabetic individuals with HF [14], which was substantially higher than that in the general population (7.1 per 1000 person-years) [15]. These associative findings are still insufficient to claim that HF plays a role in the development of T2D and further studies are needed to better dissect the HF-T2D bidirectional interplay. The coexistence of T2D and HF worsens the overall prognosis: diabetes is a predictor of poor clinical outcome, cardiovascular morbidity and mortality in HF cohorts [16, 17] and, reciprocally, incident HF is associated with a tenfold higher mortality risk in patients with T2D aged 65 years or older [18].

Furthermore, it is well validated that diabetes is the leading etiology of both CKD and end-stage kidney disease (ESKD) [19]. It is estimated that half of individuals diagnosed with type 2 diabetes and one-third of those with type 1 diabetes will develop CKD during their lifetime [19], and a meta-analysis including data from more than 5 million participants estimated the relative risk to develop CKD for females and males with T2D versus those without to be 3.34 (95% CI 2.27–4.93) and 2.84 (95% CI 1.73–4.68), respectively [20].

Unsurprisingly, not only diabetes is an established risk factor for CKD, but a high prevalence of diabetes has also been described among patients with CKD, ranging from 31 to 40% [21–23]. Although few studies have evaluated incident T2D in individuals with CKD, the Chronic Renal Insufficiency Cohort (CRIC) Study found an overall T2D incidence rate of 17.8 cases per 1000-persons years, markedly higher than that in the general population [24], supporting the bidirectionality of the interplay between these two conditions.

CKD is also considered a major risk factor for CVD, including HF [25], and the risk for cardiovascular events and death increases with declining estimated glomerular filtration rate (eGFR) [26]. An analysis from the Cardiovascular Health Study provides evidence that elevated serum creatinine is an independent predictor of CVD, HF, cardiovascular- and all-cause mortality [27]. Conversely, individuals with HF have more than two-fold higher risk of incident CKD and rapid eGFR decline [28].

The interconnections among these pathological entities assume even more relevance considering that T2D is per se associated with adverse cardiovascular outcomes, though the risk is further increased when renal dysfunction coexists, leading to a ninefold increase in relative cardiovascular mortality [29]. Altogether, these epidemiological data support the existence of a multidirectional link between T2D, HF and CKD, with each one independently increasing the incidence and worsening the prognosis of the others.

## Pathophysiological mechanisms underlining the cardio-renal-metabolic connection

### Mechanistic pathways linking T2D to cardio-renal damage

A detailed description of the molecular mechanisms through which diabetes affects the cardiovascular and renal systems is beyond the scope of this review and has been provided elsewhere [30, 31]. In brief, according to the “unifying hypothesis”, in hyperglycemic states, the excessive intracellular glucose flux leads to mitochondrial superoxide production and exacerbation of oxidative stress, postulated as the primary initiating event in diabetes-induced organ damage [32]. The increased reactive oxygen species (ROS) production causes tissue damage through several mechanisms: activation of the polyol and hexosamine pathways—which exacerbate oxidative stress in a vicious circle—activation of protein kinase C (PKC), formation of advanced glycation end-products (AGEs), resulting from non-enzymatic glycation of proteins, and upregulation of their cellular receptor RAGE [33]. In turn, AGEs can damage the heart, the vessels and the kidney both directly, causing cross-linking of matrix proteins and increasing tissue stiffness, and indirectly, via interaction with their receptor RAGE, activating signaling pathways that alter cellular function and promote oxidative stress, inflammation, and fibrosis [34]. Thus, AGEs are involved in the pathogenesis of diabetes-related organ damage, such as diabetic cardiomyopathy, diabetic kidney disease (DKD) and atherosclerosis [30, 34, 35]. AGEs and ROS are also closely associated with endothelial dysfunction, which is a major driver of diabetic microvascular and macrovascular complications [6].

Additionally, hyperglycemia is associated with the activation of local renin–angiotensin–aldosterone system (RAAS) in the myocardium and in the kidney*,* promoting vasoconstriction, fibrosis, and exacerbation of organ dysfunction [36, 37].

Furthermore, diabetes is perceived as a state of “nutrient abundance”, characterized by an aberrant activation of nutrient-sensing pathways, such as AMPK, sirtuins and mTOR, downregulating cytoprotective responses and promoting organ impairment [38, 39]. It has been demonstrated that, in glomerular podocytes, the cells responsible for the integrity of the glomerular basement membrane (GBM) and the correct functioning of the glomerular capillary loop, mTOR activation recapitulates many characteristics of DKD, such as proteinuria and mesangial expansion [40].

In parallel to glucotoxicity, insulin resistance is associated with a cellular metabolic shift towards free fatty acid (FFA) oxidation, which is more oxygen-consuming than glucose oxidation. This leads to impaired metabolic flexibility and reduced energetic efficiency, which are findings of diabetes–associated organ alterations [4, 9]. The increased uptake of FFA, when excessive, leads to accumulation of intracellular triacylglycerols, promoting oxidative stress, lipotoxicity and apoptosis [12]. In the heart, epicardial adipose tissue (EAT), a visceral fat depot located between the myocardium and the epicardium endowed with paracrine properties of regulating the myocardium and coronary arteries [41], has been proposed to function as a buffer to provide energy for the myocardium while protecting it from FFA overload [42]. T2D has been associated with pathological changes in EAT volume [43], cytokine secretory profile [44], and FFA release [45], which are potential drivers of diabetes-associated cardiovascular dysfunctions, such as atherosclerosis, intramyocardial fatty infiltration, cardiac remodeling, and HF [46].

In addition to activating several pathways driving tissue damage, diabetes compromises tissue repair, at least in part by jeopardizing the contribution of bone marrow-derived hematopoietic stem/progenitor cells (HSPCs).

Solid evidence shows that T2D is associated with a reduction in the levels of circulating HSPCs [47], mainly driven by an impaired mobilization from the bone marrow (BM) [48]. The putative HSPC property of maintaining tissue homeostasis by contributing to vascular and tissue repair [49] can explain why their shortage promotes multi-organ damage [50]. Indeed, HSPC defect has been extensively linked to the development of microvascular and macrovascular complications in T2D population [51–53], and represents a risk factor for adverse cardiovascular outcomes and death [54]. The evidence that BM-derived cells contribute to renal parenchymal regeneration after damage [55] may point to a role of HSPC shortage in DKD. HSPCs might be particularly relevant for non-albuminuric DKD, given the association between HSPCs and several CVD risk factors involved in the development of this CKD phenotype [49, 56]. In this view, diabetes can be considered a disease of impaired damage control, with defects in the physiological processes of tissue repair [57].

Finally, it should be mentioned that several other mechanisms can impair cardio-renal function in T2D, including functional abnormalities driven by metabolic and hemodynamic impairment. Insulin resistance per se leads to peripheral microvascular dysfunction [58] and skeletal muscle dysfunction [59], both of which are linked to an increased risk of HF [60, 61]. In the kidney, hyperfiltration has long been considered the major early functional alteration paving the way to the subsequent development of later DKD stages [62], though its role has been recently challenged, especially in T2D [63].

### Global impact of T2D on the cardiovascular system

Diabetes affects the cardiovascular system in different ways. It is strongly associated with the development of atherosclerosis, whose manifestations include coronary artery disease (CAD), peripheral artery disease (PAD) ad stroke. As aforementioned, hyperglycemia is closely linked with endothelial dysfunction, vascular abnormalities and inflammation, which are drivers for atherosclerotic plaque formation and progression [64]. Moreover, T2D coexists with well-known cardiovascular risk factors, including atherogenic dyslipidemia, characterized by high levels of small-dense LDL and low levels of HDL cholesterol [65]. LDL glycation occurring in hyperglycemic states increases their atherogenic potential, as glycated LDL are recognized by a scavenger receptor expressed on macrophages, resulting in a non-regulated intracellular cholesterol accumulation and enhanced plaque formation [66]. Diabetes is also associated with hypertension due to a predominance of endothelial vasoconstrictive over vasodilation signals in the diabetic milieu, a comorbidity that rises the risk of atherosclerosis and cardiovascular adverse outcomes [6]. Another frequent comorbidity which extensively contributes to the increased cardiovascular risk in T2D is obesity, which negatively impacts the cardiovascular system via different mechanisms, such as altered hemodynamic load, neurohormonal disturbances and low-grade systemic inflammation [67].

HF is a prominent diabetes-induced complication, and its onset can be promoted through different mechanisms. Atherosclerosis in the coronary arteries is strongly accelerated by diabetes, and plaque complications leading to myocardial ischemia can result in ischemic cardiomyopathy that eventually culminates in HF [68]. In parallel, metabolic alterations associated with diabetes can directly affect myocardial performance. Diabetic cardiomyopathy is defined as a diastolic or systolic dysfunction in the presence of a history of long-standing and/or poorly controlled diabetes with the exclusion of other causes of cardiomyopathy, such as coronary, congenital, valvular, and hypertensive heart disease [4, 68]. Diabetic cardiomyopathy can assume two different phenotypes: (i) restrictive, HFpEF-like phenotype, more commonly found in women with obesity and linked to coronary endothelial inflammation; (ii) dilated, HfrEF-phenotype, more closely associated with cardiomyocyte loss [69]. Although it is currently uncertain which factors are crucial for the development of one or the other phenotype, hyperglycemia, hyperinsulinemia, lipotoxicity and coronary endothelial dysfunction play a pivotal role in the development of diabetic cardiac abnormalities [4]. AGEs, maladaptive calcium homeostasis, and activation of the local RAAS, together with myocardial energetic inefficiency induced by insulin resistance, promote the development of impaired contraction, myocardial stiffness and fibrosis, which contribute to cardiac dysfunction in diabetes [35, 68, 70, 71].

### Impact of T2D on kidney function

The kidney is a major target of microvascular diabetic damage and DKD is associated with adverse health outcomes and high mortality burden. The scientific community has moved from the concept of “*diabetic nephropathy*”—defined by a rise in urinary albumin excretion classically followed by a progressive decline in renal function and traditionally classified in five stages [72]—to the term “*diabetic kidney disease”,* including all possible renal abnormalities occurring in diabetes [6].

DKD is the renal manifestation of the same hyperglycemia-induced damage pathways that target susceptible sites elsewhere in the body when exposed to the gluco- and lipotoxic diabetic milieu [38]. One of the earliest alterations in the diabetic kidney is glomerular hyperfiltration, whose pathogenesis has been linked to both altered tubule-glomerular feedback and glomerular hemodynamic abnormalities occurring in the diabetic milieu [73]. Persistent hyperfiltration results in progressive and irreversible damage to the nephron and eGFR decline, eventually terminating in ESKD. Renal tissue impairment is reflected by albuminuria and proteinuria, which are associated with functional and structural cellular alterations favored by dysregulated metabolic conditions [74]. In detail, tubular cells undergo maladaptive hypertrophy and hyperplasia in consequence of the increased glucose load delivered to the tubule and upregulate their sodium-glucose cotransporters to favor its reabsorption [75]. Consequently, the reduced amount of sodium delivered to the *macula densa* activates tubulo-glomerular feedback, which results in local activation of RAAS, hyperfiltration, and progressive damage to the glomeruli [31]. At later stages, tubular cells undergo atrophy, and their dysfunction leads to impaired protein reuptake and albuminuria [75], being tubulointerstitial fibrosis the final shared pathway for progressive renal impairment in DKD [38].

Podocytes undergo pathological changes as well, including de-differentiation, detachment, and foot-process effacement [38]. As podocytes control GBM matrix turnover, it is plausible that dysfunctional podocytes favor GBM thickening and alter its function, promoting glomerular damage and albuminuria [76]. Concurrently, the mechanical stress caused by accumulation of ECM proteins contributes to glomerular injury [77].

Mesangial cells constitute another target of diabetes-induced damage: they become hypertrophic, proliferate, and increase the synthesis of matrix proteins, leading to some of the typical structural features of diabetic glomerulopathy [78]. Thus, although hyperglycemia may be the primary initiating event in DKD, its pathogenesis is multifactorial, with diverse hemodynamic, mechanical, and structural processes contributing to the decline in kidney function [79]. Indeed, it should be mentioned that, in addition to hyperglycemia, several cardiovascular risk factors, including hypertension, obesity, hyperuricemia and inflammation, can promote renal injury in T2D [80]. Thus, these multifaceted mechanisms may sustain the development of the emerging “non-albuminuric DKD” phenotype, whose pathogenesis hypothetically relies on mechanisms operating at the macrovascular and tubule-interstital level [81], in contrast to the typical glomerular damage characterizing the classical albuminuric DKD [38].

### The bidirectional cardio-renal interplay

**Fig. 2:**
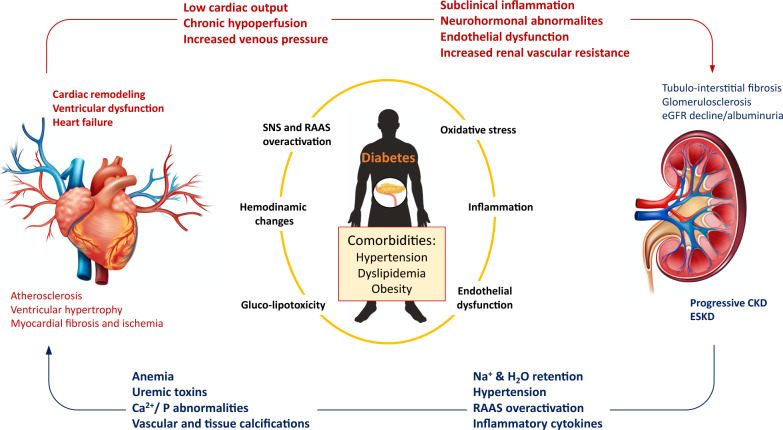
Cardio-renal interplay in the context of T2D. The figure illustrates some of the mechanisms that sustain the bidirectional relationships between kidney disease and cardiac remodeling leading to heart failure in the context of type 2 diabetes. SNS, sympathetic nervous system; RAAS, renin-angiotensin-aldosterone system; eGFR: estimated glomerular filtration rate CKD, chronic kidney disease; ESKD: end-stage kidney disease

There is broad evidence of a close interconnection between kidney and heart disease: the term cardiorenal syndrome (CRS) has been defined to underline bidirectionality of heart-kidney interplay, with acute or chronic dysfunction of one organ leading to acute or chronic dysfunction of the other [82], conferring relevant morbidity and mortality [83]. Since combined heart and kidney abnormalities can differ in their clinical presentation and time frame (acute vs chronic), a subdivision of CRS in five subtypes has been adopted [82]. A detailed discussion of pathophysiological mechanisms potentially responsible for CRS is beyond the scope of this article as such information can be found elsewhere [82–84]. It is worth mentioning that hemodynamic and neurohormonal abnormalities are putatively key players in the detrimental crosstalk between the failing heart and the failing kidney [83]. Briefly, HF-associated low cardiac output, effective hypovolemia, and excess of vasoconstrictive mediators lead to chronic renal hypoperfusion and decrease in eGFR, favoring CKD initiation and/or progression [82]. Conversely, sodium and water retention and chronic RAAS activation in CKD exacerbate hypertension and increase cardiac pre- and after-load. These hemodynamic abnormalities—together with CKD-associated uremic toxins retention and chronic inflammation—sustain pathological cardiac remodeling and the onset and worsening of cardiac dysfunction, completing a vicious cycle injurious to both organs [82]. The parallel pathways leading to cardiac and renal disease in the context of T2D are illustrated in Fig. 2.

### Mechanisms for the impact of cardiovascular dysfunction on T2D onset

Not only diabetes is traditionally considered a major cardiovascular risk factor, but, conversely, there is growing evidence that cardiovascular dysfunction can promote metabolic alterations and new-onset T2D. To explain this relationship, different mechanisms have been proposed, although not entirely elucidated. First, endothelial dysfunction has increasingly been recognized as a common soil between disorders of glucose and cardiovascular homeostasis [85]. This hypothesis is supported by a close bidirectional relationship between insulin resistance, a core metabolic abnormality in T2D, and endothelial dysfunction, which is one of the crucial initiating events in the pathogenesis of CVD [86]. Additionally, HF can per se be considered an “insulin resistant state” [87, 88]. One mechanism affecting insulin sensitivity in HF is neuro-hormonal activation [89, 90]: in particular, sympathetic nervous system (SNS) hyperactivation that occurs in HF impairs glucose homeostasis via stimulation of alpha-adrenergic receptors, resulting in skeletal muscle hypoperfusion and diminished tissue glucose uptake [91]. Insulin resistance is not the only mechanism through which catecholamine hypersecretion affects glucose homeostasis in HF: chronic SNS activation enhances lipolysis, and elevated FFA can deposit ectopically and have been shown to promote hepatic gluconeogenesis and impair insulin secretions from pancreatic β-cells [92].

The onset of T2D in individuals with HF may also be mediated by the overactivity of the RAAS: angiotensin II (AngII) induces skeletal muscle vasoconstriction and defective muscle glucose uptake, which are associated with diminished insulin sensitivity [93]. AngII has also been shown to directly interfere with insulin signaling pathway by inducing phosphorylation of insulin signal molecules, thereby inhibiting downstream signal transduction [94]. Additionally, AngII can per se induce β-cell dysfunction via an endoplasmic reticulum stress-mediated mechanism [95], impairing insulin secretion and favoring β-cell apoptosis [96].

Other neuro-hormonal factors involved are natriuretic peptides (NPs): they have recently emerged as metabolic hormones, improving insulin sensitivity, lipid oxidation and browning of the adipose tissue [97]. Additionally, there is evidence that NP exert a direct influence on β-cells, modulating their function and enhancing insulin secretion [98]. Metabolic dysregulated conditions, such as obesity and T2D, are associated with low NP levels, suggesting that NP defect may impair glucose homeostasis [99]. Although NP serum levels are increased in HF, their effectiveness is reduced [100]: this evidence suggests that HF is paradoxically similar to metabolic diseases characterized by NP deficiency, pointing to a role of NP in the development of HF-associated glucose abnormalities.

It is also broadly recognized that proinflammatory cytokines play a role in the development of insulin resistance by interfering with insulin signaling [101]. HF is a pro-inflammatory condition, and a significant association between inflammation-related biomarkers in HF and new-onset T2D has been described, suggesting that immuno-inflammatory mechanisms may be involved in the pathogenesis of HF-associated diabetes [102].

The influence of physical activity on T2D onset has been widely described across literature [103, 104], and exercise activity limitation experienced by patients with HF potentially contributes to the development of glucose abnormalities in this condition [89]. HF is associated with reduced skeletal muscle perfusion and loss of muscle mass, the main tissue where glucose is utilized, and both these processes lead to impaired peripheral glucose utilization and diminished sensitivity to insulin [90].

### Mechanisms for the impact of kidney dysfunction on T2D onset

While diabetes is a prominent risk factor for kidney disease, it becomes to be appreciated that kidney disease may, in turn, promote metabolic dysregulation and new-onset or worsening T2D. Although some studies suggest that the incident rate of T2D in patients with CKD is significantly higher than that in the general population [24, 105], the pathophysiology underlying glucose abnormalities in CKD remains largely unclear.

There is evidence that insulin resistance is an early finding in people diagnosed with CKD [106], and kidney dysfunction seems to be associated per se with defective insulin-signaling pathway, since insulin resistance is a frequent abnormality in CKD regardless of its etiology [107]. The skeletal muscle is recognized as the primary site of CKD-associated insulin resistance, which is caused by “post-receptor defects” involving signal transduction proteins [107]. Several mechanisms are potentially involved in the genesis of CKD-associated glucose abnormalities. CKD is a condition characterized by chronic inflammation and enhanced oxidative stress [108], and it is well validated that proinflammatory cytokines promote insulin resistance through post-translational modifications of signal-transduction proteins [107, 109].

Furthermore, metabolic acidosis—a common CKD complication—has been associated with decreased sensitivity to insulin in both healthy and CKD individuals [110, 111]. Although only few studies have been conducted, there is evidence that acidosis correction by administration of sodium bicarbonate ameliorates insulin resistance, supporting the causal involvement of metabolic acidosis in suboptimal biological response to insulin [112].

Another mechanism potentially affecting glucose homeostasis in CKD is vitamin D deficiency, a very frequent finding in individuals with impaired kidney function [113]. Vitamin D status may directly influence glucose metabolism: vitamin D regulates insulin release through the modulation of intracellular calcium in β-cells [114], increases the expression of the insulin receptor [115], and its deficiency is associated with secondary hyperparathyroidism, which can diminish insulin secretion [116]. A body of evidence supports the existence of an association between vitamin D deficiency and glucose metabolism abnormalities in populations with CKD [117, 118]. A randomized controlled-study including non-diabetic individuals with CKD found that insulin resistance incidence was significantly higher in the vitamin D-deficient than in the vitamin D-normal group; in the same study, the supplementation with activated vitamin D analogs significantly ameliorated insulin sensitivity and β-cell function, supporting the hypothesis of a direct role of vitamin D in regulating metabolic homeostasis in CKD [119].

Other factors favoring abnormalities in glucose metabolism are toxins accumulating as the renal function critically diminishes, such as blood urea, p-cresyl sulfate, and asymmetric dimethylarginine [120–122]. Although these compounds can alter glucose homeostasis via inflammation-mediated insulin resistance [107], urea can directly induce β-cell dysfunction: elevated levels of urea increase islet protein O-GlcNAcylation and impair glycolysis, resulting in insulin secretory defects [123].

Understanding the pathogenesis of CKD-associated glucose abnormalities becomes even more crucial considering that CKD is a condition associated with high cardiovascular risk [8] and insulin resistance is a predictor of CVD and cardiovascular mortality in several CKD cohorts [124, 125].

## Integrated management of CMR disease

Since T2D, HF and CKD share a common pathophysiologic background and often coexist, adopting a holistic therapeutic strategy targeting CMR comorbidities would have a synergistic effect on patient health, resulting in significant outcome improvements. Data from large-scale clinical trials have consistently shown that the beneficial effects of novel glucose-lowering drugs, such as sodium-glucose cotransporter 2 inhibitors (SGLT2i) and glucagon-like peptide 1 receptor agonists (GLP-1 RA), extend far beyond glycemic control, reducing important cardiovascular and renal endpoints in populations with T2D [126–128]. Similarly, the selective, non-steroidal mineralocorticoid receptor antagonist Finerenone has exhibited cardiorenal protective effects in individuals with T2D and CKD [129, 130]. The CMR abnormalities on which these classes of drugs have been shown to have favorable impact are summarized in Table 1. The putative mechanisms responsible for CMR benefits of these medications have been deeply revised elsewhere [131–135]. Briefly, while SGLT2i are believed to exert cardiorenal protection prominently via a hemodynamic action sustained by their natriuretic and osmotic effects [136], anti-atherogenic and immune-modulating mechanisms may be responsible for GLP-1 RA-mediated protective effects [133, 137]. The novel bireceptor agonist Tirzepatide simultaneously activates two incretin-dependent pathways [138], and this duality acts synergistically on glycemic and weight control, significantly improving metabolic outcomes when compared to selective GLP-1 RA [139]. On the other hand, Finerenone exerts its cardiorenal protection at a different level, targeting MR overactivation, a major pro-inflammatory and pro-fibrotic driver of cardiorenal complications in T2D [135].

**Table 1:** Medications with proven CMR benefits and their respective targeted CMR abnormalities. CMR, cardio-metabolic-renal; T2D, type 2 diabetes; HF, heart failure; CKD, chronic kidney disease

	Metabolic targets	Cardiovascular targets	Renal targets
SGLT2i	• T2D [128, 132, 136] • Overweight [132, 136]	• Hypertension [132, 136] • HF [127, 134, 136] • CV death [128, 132]	• CKD [127, 131, 132, 142] • ESKD [127] • Albuminuria [132, 142]
GLP1 – RA	• T2D [126, 131] • Overweight/obesity [126, 131]	• Hypertension [126, 134, 137] • Atherosclerosis [131, 137] • Stroke [126, 134, 137] • CV death [131, 137]	• Albuminuria [126, 133, 134] • CKD? [131, 134]
Finerenone	• T2D (?) [129, 130]	• Hypertension [130] • HF [129, 130, 135]	• CKD [129, 135]
Tirzepatide	• T2D [138, 139] • Overweight/obesity [138, 139]	• CV risk factors [138, 143]	• Albuminuria? [144] • CKD? [144]

While SGLT2i are now popular drugs for the management of T2D, they appear to exert their beneficial effects against HF and ESKD largely independently from glycemic control. Indeed, protection from hospitalization for HF, as well as from the progression of CKD have been demonstrated even in people without diabetes. In addition, the glucose lowering capacity of SGLT2i reduces together with the decline in renal function, though the protection against ESKD is preserved until the later CKD stages [140]. On the other side, the cardiovascular or renal benefits of GLP-1RA in non-diabetic individuals still needs to be demonstrated and mediation analyses found the change in HbA1c to be a predictor of the end-organ protection [141].

The use of single glucose-lowering agents with manifold protective effects on CMR system, as well as concomitant treatment with multiple drugs having complementary mechanisms of action are promising for the management of CMR disease spectrum. Thus, there is potential to use multifactorial intervention to fully take advantages of complementary pharmacological effects and simultaneously target comorbid conditions.

## Conclusions

A large amount of epidemiological data from observational and clinical trials supports the existence of a substantial overlap between metabolic, cardiovascular, and renal diseases, with the onset of one increasing the risk and worsening the outcome of the others. These three entities share common pathophysiological mechanisms, whose activation results in a vicious cycle of perpetuation of diseases processes, increasing morbidity and mortality. The identification of the pathophysiological interconnections among these comorbidities is key to unravel common therapeutic approaches. A better understanding of the shared core mechanisms underlining CMR disease can provide targets for pharmacological intervention aiming at interrupting the detrimental crosstalk, thereby ameliorating clinical outcomes.

Adopting a tailored therapeutic approach addressing overall patient’s comorbid conditions becomes even more essential considering the availability of novel glucose-lowering drugs with proven renal and cardiovascular protection. Further research is needed to gain deeper insight into CMR pathophysiology, elucidate the benefits from an integrated management of comorbid T2D, CVD and CKD and guide individualized treatment choices in clinical practice (Table 2).

**Table 2:** Current unmet needs and potential future directions to improve CMR management

Unmet needs	Future directions
Novel medications with CMR benefits	• Identification of druggable CMR connectors • Further efficacy and safety data of novel therapies
Combination therapy strategy	• Better understanding of drug mechanisms of action • Outcomes trials testing combination therapies
Integrated management of CMR disease	• Multidisciplinary approach
Risk-stratified personalized care	• Risk score validation for individualized prognostication • Clinical trials in specific populations (women/young/primary prevention)
Drug prioritization	• Head-to-head drug comparative trials

CMR, cardio-metabolic-renal

## Data Availability

All the data used to write this manuscript are presented in the text, tables or references.
